# Supplementary material for: Correlation between Mild Traumatic Brain Injury-Induced Inflammatory Cytokines and Emotional Symptom Traits: A Systematic Review
Source: Brain Sci. 2022 Jan 12;12(1):102. doi: 10.3390/brainsci12010102 (PMC8773760; doi:10.3390/brainsci12010102)
Supplement: Supplementary file 1 [file brainsci-12-00102-s001.zip › brainsci-1518351-supplementary.pdf]

Table S1: Search Strategy for Depression

EMBASE	MEDLINE	PUBMED	COCHRANE	PSYCHNFO
**Strategy**:<br><br>1. exp inflammation/ or inflammat*.mp.<br>2. exp cytokine/ or cytokine*.mp.<br>3. exp neuroinflammation/ or neuroinflammat*.mp.<br>4. 1 or 2 or 3<br>5. mtbi.mp.<br>6. exp brain concussion/ or exp concussion/ or concussion.mp.<br>7. exp mild traumatic brain injury/ or mild traumatic brain injur*.mp.<br>8. 5 or 6 or 7<br>9. exp depression/ or depression.mp. or exp long term depression/ or exp major depression/<br>10. 4 and 8 and 9<br>11. limit 11 to (human and english language).	**Strategy**:<br><br>1. exp inflammation/ or inflammat*.mp.<br>2. exp cytokine/ or cytokine*.mp.<br>3. exp neuroinflammation/ or neuroinflammat*.mp.<br>4. 1 or 2 or 3<br>5. mtbi.mp.<br>6. exp brain concussion/ or exp concussion/ or concussion.mp.<br>7. exp mild traumatic brain injury/ or mild traumatic brain injur*.mp.<br>8. 5 or 6 or 7<br>9. exp depression/ or depression.mp. or exp long term depression/ or exp major depression/<br>10. 4 and 8 and 9<br>11. limit 11 to (human and english language).	**Strategy**:<br><br>((depression) AND ((mtbi) OR (concussion) OR (brain concussion) OR (mild traumatic brain injur*)) AND ((inflammat*) OR (cytokine*) OR (neuroinflammat*))	**Strategy:**<br><br>((depression) AND ((mtbi) OR (concussion) OR (brain concussion) OR (mild traumatic brain injur*)) AND ((inflammat*) OR (cytokine*) OR (neuroinflammat*))	**Strategy:**<br><br>1. exp inflammation/ or inflammat*.mp.<br>2. exp cytokine/ or cytokine*.mp.<br>3. exp neuroinflammation/ or neuroinflammat*.mp.<br>4. 1 or 2 or 3<br>5. mtbi.mp.<br>6. exp brain concussion/ or exp concussion/ or concussion.mp.<br>7. exp mild traumatic brain injury/ or mild traumatic brain injur*.mp.<br>8. 5 or 6 or 7<br>9. exp depression/ or depression.mp. or exp long term depression/ or exp major depression/<br>10. 4 and 8 and 9<br>11. limit 11 to (human and english language).

Table S2: Search Strategy for PTSD

EMBASE	MEDLINE	PUBMED	COCHRANE	PSYCHNFO
**Strategy**:<br><br>12. exp inflammation/ or inflammat*.mp.<br>13. exp cytokine/ or cytokine*.mp.<br>14. exp neuroinflammation/ or neuroinflammat*.mp.<br>15. 1 or 2 or 3<br>16. mtbi.mp.<br>17. exp brain concussion/ or exp concussion/ or concussion.mp.<br>18. exp mild traumatic brain injury/ or mild traumatic brain injur*.mp.<br>19. 5 or 6 or 7<br>20. ptsd.mp. or exp posttraumatic stress disorder<br>21. 4 and 8 and 9<br>22. limit 11 to (human and english language).	**Strategy**:<br><br>12. exp inflammation/ or inflammat*.mp.<br>13. exp cytokine/ or cytokine*.mp.<br>14. exp neuroinflammation/ or neuroinflammat*.mp.<br>15. 1 or 2 or 3<br>16. mtbi.mp.<br>17. exp brain concussion/ or exp concussion/ or concussion.mp.<br>18. exp mild traumatic brain injury/ or mild traumatic brain injur*.mp.<br>19. 5 or 6 or 7<br>20. ptsd.mp. or exp posttraumatic stress disorder<br>21. 4 and 8 and 9<br>22. limit 11 to (human and english language).<br>23.	**Strategy**:<br><br>(((PTSD) OR (posttraumatic stress disorder)) AND ((mtbi) OR (concussion) OR (brain concussion) OR (mild traumatic brain injur*)) AND ((inflammat*) OR (cytokine*) OR (neuroinflammat*))	**Strategy:**<br><br>(((PTSD) OR (posttraumatic stress disorder)) AND ((mtbi) OR (concussion) OR (brain concussion) OR (mild traumatic brain injur*)) AND ((inflammat*) OR (cytokine*) OR (neuroinflammat*))	**Strategy:**<br><br>12. exp inflammation/ or inflammat*.mp.<br>13. exp cytokine/ or cytokine*.mp.<br>14. exp neuroinflammation/ or neuroinflammat*.mp.<br>15. 1 or 2 or 3<br>16. mtbi.mp.<br>17. exp brain concussion/ or exp concussion/ or concussion.mp.<br>18. exp mild traumatic brain injury/ or mild traumatic brain injur*.mp.<br>19. 5 or 6 or 7<br>20. ptsd.mp. or exp posttraumatic stress disorder<br>21. 4 and 8 and 9<br>22. limit 11 to (human and english language).<br>**23.**

Table S3: Search Strategy for Anxiety

EMBASE	MEDLINE	PUBMED	COCHRANE	PSYCHNFO
**Strategy**:<br><br>23. exp inflammation/ or inflammat*.mp.<br>24. exp cytokine/ or cytokine*.mp.<br>25. exp neuroinflammation/ or neuroinflammat*.mp.<br>26. 1 or 2 or 3<br>27. mtbi.mp.<br>28. exp brain concussion/ or exp concussion/ or concussion.mp.<br>29. exp mild traumatic brain injury/ or mild traumatic brain injur*.mp.<br>30. 5 or 6 or 7<br>31. anxiety.mp. or exp anxiety disorder/ or exp anxiety/<br>32. 4 and 8 and 9<br>33. limit 11 to (human and english language).	**Strategy**:<br><br>24. exp inflammation/ or inflammat*.mp.<br>25. exp cytokine/ or cytokine*.mp.<br>26. exp neuroinflammation/ or neuroinflammat*.mp.<br>27. 1 or 2 or 3<br>28. mtbi.mp.<br>29. exp brain concussion/ or exp concussion/ or concussion.mp.<br>30. exp mild traumatic brain injury/ or mild traumatic brain injur*.mp.<br>31. 5 or 6 or 7<br>32. anxiety.mp. or exp anxiety disorder/ or exp anxiety/<br>33. 4 and 8 and 9<br>34. limit 11 to (human and english language).	**Strategy**:<br><br>(((anxiety) OR (anxiety disorder)) AND ((mtbi) OR (concussion) OR (brain concussion) OR (mild traumatic brain injur*)) AND ((inflammat*) OR (cytokine*) OR (neuroinflammat*))	**Strategy:**<br><br>(((anxiety) OR (anxiety disorder)) AND ((mtbi) OR (concussion) OR (brain concussion) OR (mild traumatic brain injur*)) AND ((inflammat*) OR (cytokine*) OR (neuroinflammat*))	**Strategy:**<br><br>24. exp inflammation/ or inflammat*.mp.<br>25. exp cytokine/ or cytokine*.mp.<br>26. exp neuroinflammation/ or neuroinflammat*.mp.<br>27. 1 or 2 or 3<br>28. mtbi.mp.<br>29. exp brain concussion/ or exp concussion/ or concussion.mp.<br>30. exp mild traumatic brain injury/ or mild traumatic brain injur*.mp.<br>31. 5 or 6 or 7<br>32. anxiety.mp. or exp anxiety disorder/ or exp anxiety/<br>33. 4 and 8 and 9<br>34. limit 11 to (human and english language).
